# Hepatopathy following consumption of a commercially available blue-green algae dietary supplement in a dog

**DOI:** 10.1186/s12917-015-0453-2

**Published:** 2015-06-19

**Authors:** Adrienne C. Bautista, Caroline E. Moore, Yanping Lin, Martha G. Cline, Noemi Benitah, Birgit Puschner

**Affiliations:** California Animal Health and Food Safety Laboratory System, Davis, CA USA; Department of Molecular Biosciences, School of Veterinary Medicine, University of California, Davis, CA USA; Red Bank Veterinary Hospital, Tinton Falls, NJ USA

**Keywords:** Dog, Microcystin, Blue-green algae, Hepatopathy, Toxicosis, Dietary supplement

## Abstract

**Background:**

Dietary supplement use in both human and animals to augment overall health continues to increase and represents a potential health risk due to the lack of safety regulations imposed on the manufacturers. Because there are no requirements for demonstrating safety and efficacy prior to marketing, dietary supplements may contain potentially toxic contaminants such as hepatotoxic microcystins produced by several species of blue-green algae.

**Case presentation:**

An 11-year-old female spayed 8.95 kg Pug dog was initially presented for poor appetite, lethargy polyuria, polydipsia, and an inability to get comfortable. Markedly increased liver enzyme activities were detected with no corresponding abnormalities evident on abdominal ultrasound. A few days later the liver enzyme activities were persistently increased and the dog was coagulopathic indicating substantial liver dysfunction. The dog was hospitalized for further care consisting of oral S-adenosylmethionine, silybin, vitamin K, and ursodeoxycholic acid, as well as intravenous ampicillin sodium/sulbactam sodium, dolasetron, N-acetylcysteine, metoclopramide, and intravenous fluids. Improvement of the hepatopathy and the dog’s clinical status was noted over the next three days. Assessment of the dog’s diet revealed the use of a commercially available blue-green algae dietary supplement for three-and-a-half weeks prior to hospitalization. The supplement was submitted for toxicology testing and revealed the presence of hepatotoxic microcystins (MCs), MC-LR and MC-LA. Use of the supplement was discontinued and follow-up evaluation over the next few weeks revealed a complete resolution of the hepatopathy.

**Conclusions:**

To the authors’ knowledge, this is the first case report of microcystin intoxication in a dog after using a commercially available blue-green algae dietary supplement. Veterinarians should recognize the potential harm that these supplements may cause and know that with intervention, recovery is possible. In addition, more prudent oversight of dietary supplement use is recommended for our companion animals to prevent adverse events/intoxications.

## Background

Dietary supplement use to augment overall health in humans is not a new phenomenon. More than half of all adults in the United States currently take one or more dietary supplements [1]. Extension of this practice to household pets is also commonplace. More than a fourth of all households in one study reported feeding supplements to their dogs [2]. More alarming is that the dietary supplement industry is largely self-regulated, although there are some safeguards in place following the passage of the 1994 Dietary Supplement Health and Education Act (DSHEA) by the United States Food and Drug Administration (FDA) [3]. Good manufacturing practices and adverse event reporting are just a few of the safeguards; however, there is no premarket review of safety [4]. Thus, many consumers falsely assume that dietary supplements are screened for safety prior to marketing. Reports of contamination with various adulterants including microcystins [5, 6] highlight the need for vigilance in daily use of these products in humans and animals.

Dietary supplements containing blue-green algae have been marketed to the public since the early 1980’s [7]. *Spirulina platensis* and *Aphanizomenon flos aquae* (*A. flos aquae*) are two of the most common species either grown under controlled conditions or collected from the natural environment. In the case of *A. flos aquae*, the Upper Klamath Lake in Oregon is one of the most used sources for harvesting these blue-green algae. Unfortunately, the open, natural environment also allows other potentially toxic cyanobacteria, such as *Microcystis aeruginosa* to flourish. Thus, contamination of the *A. flos aquae* during harvesting can and does occur [8]. Furthermore, contamination of dietary supplements containing naturally harvested *A. flos aquae* with microcystins has been documented [5, 7, 9] and poses a health risk to the consumer.

Microcystins are potent cyclic peptides produced by certain species of cyanobacteria and are quite resistant to degradation. Currently, over 100 congeners have been identified [10, 11], with MC-LR (abbreviated for common amino acids leucine and arginine at positions 2 and 4, respectively) [12] being the most consistently identified and potentially potent congener. MC-LR and several other congeners are known to inhibit protein phosphatase leading to acute liver failure [13]. However, due to their diverse nature, a complete understanding of the acute and chronic effects of the MCs is lacking. Congeners other than MC-LR have been linked with animal intoxications [14] and effects on other organs have been noted [15] raising the possibility that human and animal risk may be underestimated. Intoxication or adverse effects from microcystin contaminated dietary supplements have not been reported in humans or animals. The current case report describes the discernible clinical and toxicological findings in a dog following the use of a commercially available blue-green algae dietary supplement and illustrates the risks involved when using unregulated over-the-counter products.

## Case presentation

An 11-year-old female spayed Pug dog, weighing 8.95 kg with a body condition score of 6/9, initially was presented to her veterinarian for poor appetite, lethargy, polyuria, polydipsia, and an inability to get comfortable. Serum biochemical abnormalities included high alkaline phosphatase (ALP) (414 U/L, reference (ref) range 23–212 U/L) and alanine aminotransferase (ALT) (>1000 U/L, ref range 10–100 U/L) activities (Table 1) and subsequently, the dog was referred to Red Bank Veterinary Hospital (RBVH) (New Jersey) for an abdominal ultrasound. Additional abnormalities found at initial presentation included thrombocytosis (527 K/μL, ref range 175–500 K/μL) and isosthenuria (1.007, ref range 1.015-1.050). Five months prior to presentation, the dog’s liver enzyme concentrations were within the reference range (Table 1). Past medical history included a rectal sarcoma excised approximately 32 months prior followed by chemotherapy (4 treatments of doxorubicin) with no evidence of recurrence at the time of presentation. In addition, the dog was treated for pancreatitis as an outpatient 5 months prior; pancreatitis was diagnosed by abdominal ultrasound and results of serum canine pancreas-specific lipase. Furthermore, three months prior to presentation, the dog was treated for urolithiasis by a cystotomy (struvite). The dog had concurrent problems including a collapsing trachea (managed with hydrocodone 1.25 mg, PO, q 12 h), keratoconjunctivitis sicca (treated with ophthalmic tacrolimus aqueous 0.02 % solution 1 drop, OU, q 12 h and artificial tears), and urinary incontinence (controlled with phenylpropanolamine 25 mg, PO, q 12 h). The owner prepared home-cooked meals for the dog since the episode of pancreatitis 5 months prior which included a combination of cooked chicken breast, turkey breast, or ground beef with peas, carrots, and white potato. On initial physical examination, the dog was alert, eupneic, dehydrated, and the abdomen palpated as non-painful. Stertor and pigmentary keratitis, common to this breed, were also noted. In-house assessment of ALT activity was significantly high (2620 U/L, ref range 10–100 U/L). On abdominal ultrasound, the liver appeared diffusely normal with smooth margins. There was no evidence of pancreatitis or free abdominal fluid. Therefore, no cause for the markedly high ALT activity was identified. The owner elected outpatient care consisting of the administration of PlasmaLyte A (200 ml, SC, q 24 h), amoxicillin trihydrate/clavulanate potassium (125 mg, PO, q 12 h) and maropitant (8 mg, PO, q 24 h). Three days later, the dog was presented again to RBVH for continued lethargy and inappetence, as well as one episode of vomiting. Serum biochemistry abnormalities included high ALT, ALP and aspartate aminotransferase (AST) activities (Table 1), prompting assessment of coagulation. Both prothrombin time (PT) and partial thromboplastin time (PTT) were significantly prolonged at 41 sec and >300 sec, respectively (ref range 11–17 s and 72–102 s). The dog was hospitalized for supportive care due to her clinical signs, serum biochemical abnormalities and coagulopathy. Supportive care was initiated consisting of S-adenosylmethionine with silybin (225 mg with 24 mg, PO, q 24 h), ursodeoxycholic acid (90 mg, PO, q 24 h), ampicillin sodium/sulbactam sodium (195 mg, IV, q 8 h), dolasetron (5.3 mg, IV, q 24 h), N-acetylcysteine (140 mg/kg loading dose; 50 mg/kg, IV, q 6 h ×3 days), metoclopramide (1 mg/kg/day, IV, CRI), vitamin K1 (26 mg, SC, q 24 h) once daily and IV fluid therapy, consisting of PlasmaLyte A supplemented with B-complex vitamins. Clinical and serum biochemical improvement of the hepatopathy were noted over the next 3 days of hospitalization (Table 1).

**Table 1:** Serial serum biochemistry and coagulation panel results at various time points after initial presentation^a^

Parameter	5 months prior	Day 1	Day 4	Day 5	Day 6	Day 7	Day 14	Day 40	Reference Range
ALT (U/L)	50	2620	2054	838	704	620	181	42	10-100
AST (U/L)			503	208	167	192	50	39	0-50
ALP (U/L)	110	414	753	277	402	640	308	119	23-212
Total bilirubin (mg/dL)	0.4		1.1	1.4	0.8	0.6	0.4	0.4	0-0.9
PT (sec)			41			16	14		11-17
PTT (sec)			>300			97			72-102

^a^ALT = alanine aminotransferase; AST = aspartate aminotransferase; ALP = alkaline phosphatase; PT = prothrombin time; PTT = partial thromboplastin time

**Fig. 1:**
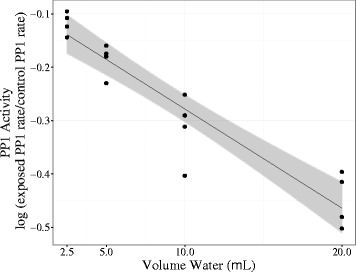
Change in PP1 activity. One gram of blue-green algae supplement powder was dissolved in water and sonicated on ice, centrifuged, and supernatant was used to determine the presence of protein phosphatase 1 (PP1) inhibitors. The y-axis is the log (exposed PP1 rate/control PP1 rate) and the x-axis is the volume of extracted water from the powder in μL tested. Each protein phosphatase inhibition assay (PPIA, n = 4), analyzed in triplicate with a substrate blank, is represented as one point, and the linear model with a 95 % confidence interval is displayed with the data. As the amount of extract increases, the activity of PP1 significantly decreases compared to control, with a p value of 5.9 × 10^−8^

Scrupulous assessment of the dog’s home prepared diet revealed the use of a dietary supplement for the previous three-and–a-half weeks. The supplement consisted of 100 % certified organic *Aphanizomenon flos aquae* and a suggested dose for a dog weighing 13.6 kg and under was 1 of the provided measurement scoop (equal to 1 gram) of powder one or more times daily. The dog’s owner had been administering less than a 1 scoop of the supplement once daily. The owner was additionally providing a veterinary glucosamine and chondroitin supplement per manufacture recommendations. The powdered blue-green algae dietary supplement was submitted for toxicology testing due to concern for the possible presence of hepatotoxic microcystins. To evaluate the functional toxicity of the powder, a protein phosphatase inhibition assay (PPIA) was performed after one gram of powder was dissolved in water, sonicated, and centrifuged to obtain a crude extract. To determine if an increasing dose of powder resulted in a dose-dependent increase in toxicity (protein phosphatase inhibition) PPIA data was log transformed and analyzed by linear regression using the statistical software R. As concentrations of powder extract were increased, protein phosphatase activity significantly decreased (slope −0.0186, p < 0.001, Fig. 1). To evaluate the presence of 4 specific MC congeners for which analytical standards are available (MC-LR, −LA, −RR, −YR), samples of powder were extracted using methanol and analyzed by liquid chromatography-mass spectrometry/mass spectrometry. The powder contained 166 ng/g of MC-LR and 962 ng/g of MC-LA; none of the other MC congeners were detected in the powder.

Use of the blue-green algae supplement was discontinued just prior to hospitalization. The dog was sent home after 4 days of hospitalization (Day 7) on amoxicillin trihydrate/clavulanate potassium (125 mg, PO, q 12 h × 7 days), vitamin K1 (25 mg, PO, q 24 h × 5 days), S-adenosylmethionine with silybin (225 mg with 24 mg, PO, q 24 h), vitamin E (200 IU, PO, q 24 h), ursodeoxycholic acid (90 mg, PO, q 24 h × 7 days), and milk thistle (150 mg, PO, q 24 h). While hospitalized, the dog consumed a combination of canned or boiled chicken breast with white rice and was discharged with recommendations to continue these foods short term before transition to a complete and balanced home prepared maintenance diet. Two weeks following discharge, the dog was started on a complete and balanced home prepared diet (34 % metabolizable energy (ME) protein, 19 % ME fat, 47 % ME carbohydrate) using ingredients such as cooked chicken breast, turkey breast, or ground beef with white potato, and mixed vegetables formulated by a board-certified veterinary nutritionist. Follow-up evaluation over the next few weeks revealed a complete resolution of clinical signs and serum biochemical abnormalities (Table 1) and therefore, the use of liver support supplements (S-adenosylmethionine with silybin, milk thistle, and vitamin E) was discontinued. Urine analysis 5 months following discharge revealed improved urine concentrating ability (urine specific gravity 1.017). At approximately one-year follow-up, the dog was healthy with no recurrence of clinical signs or laboratory abnormalities indicative of hepatic disease or dysfunction.

## Discussion

The current case study confirms a case of microcystin poisoning in a dog following the use of a blue-green algae dietary supplement and highlights the importance of the toxic potential of these dietary supplements. Blue-green algae such as *A. flos aquae* marketed as dietary supplements are commonly grown in open, natural environments which allows for possible contamination with toxin-producing cyanobacteria. *A. flos aquae* has the ability to produce a multitude of toxins including anatoxin-a, saxitoxins, cylindrospermospin, and BMAA, in addition to microcystins [5]. More importantly, microcystins have been identified as common contaminants of algae dietary supplements [5]. With over 100 MC congeners identified and named according to variable amino acids positions, complete evaluation of supplements or algal material for all congeners is not feasible [10, 11]. Current methods for detecting MCs range from simple biological based screening methods to more complex analytical techniques such as mass spectrometry [16]. Many of the commonly used biological detection methods are not congener specific and therefore may not accurately reflect the true toxic nature of the sample being tested. In addition, the ability of these techniques to identify all the various MC congeners is hampered by the lack of standard reference materials for the toxins. The most consistently identified microcystin, MC-LR, is also believed to be the most potent congener. MC-LR was identified in the supplement along with MC-LA. MCs cause hepatic necrosis by covalently binding to the catalytic domains of protein phosphatases type 1 and 2A, ultimately inhibiting their activity [17]. Hepatic necrosis following exposure to microcystins has been documented in humans, mice, rats, guinea pigs, sheep, cattle and dogs [18–22]. Post-mortem, MCs poisoning is characterized by severe gross hepatomegaly with progressive centrilobular hepatocyte necrosis, rounding and dissociation. Subsequent breakdown of the sinusoidal endothelium results in intrahepatic hemorrhage and rapidly ensuing death [20]. Histopathology was not performed in this case because the dog was coagulopathic. The dramatic increase in ALT activity and development of a coagulopathy following a few weeks of exposure to the dietary supplement support the diagnosis of a hepatopathy and likely underlying hepatic necrosis. Fortunately, there was complete resolution of these changes after cessation of supplement exposure indicating that the damage is reversible if recognized before complete hepatic failure ensues.

Although a vast amount of information exists on the acute toxicity of microcystins, chronic toxicity data is lacking. The acute, single dose LD_50_ for the various microcystins ranges from 43 μg/kg to 11 mg/kg depending on the congener, species affected and route of administration. For example, the oral LD_50_ for MC-LR in mice is 10.9 mg/kg versus the intraperitoneal LD_50_ of 43 μg/kg [23, 24]. Most of the commonly identified congeners, such as MC-LA and MC-YR, have a similar LD_50_ range as MC-LR. However, when analyzing for the presence of microcystins in a diagnostic case, not all the congeners are tested for. Thus, the true toxicity of the microcystins may be underestimated. In the current case, the supplement was analyzed for the presence of microcystins LR, LA, YR and RR. Both MC-LR and MC-LA were identified with an estimated amount of 166 ng/g MC-LR and 962 ng/g MC-LA. Giving the recommended 1 scoop (1 gram) containing 166 ng/g of MC-LR and 962 ng/g of MC-LA, an 8.95 kg dog would consume on average 0.0185 μg/kg/day of MC-LR and 0.107 μg/kg/day of MC-LA. Total daily microcystin intake from supplement use was estimated to be 0.126 μg/kg/day. The World Health Organization has set the tolerable daily intake for human ingestion of MC-LR at 0.04 μg/kg/day [25], yet no such tolerance exists for canines or other animals. The presence of high concentrations of MC-LA, whose hepatotoxicity is similar to MC-LR, contributed significantly to the development of the hepatopathy in this dog. In addition, due to the limitation of available reference standards for other microcystin congeners, it is conceivable that the supplement contained congeners other than MC-LR and MC-LA.

Increased serum activities of enzymes indicative of hepatocellular damage have been noted in both acute and chronic microcystin toxicity studies [20, 26–29]. In addition, prolonged sub-lethal exposure to MC-LR has been shown to result in several hepatotoxic effects including increased serum activity of hepatic enzymes, decreased hepatic protein phosphatase type 1 and 2A activity and histologically, hepatocellular vacuolization and apoptosis [26]. The dog in the current case also showed biochemical evidence indicative of hepatocellular damage and dysfunction likely induced by prolonged exposure to microcystins in the dietary supplement. On Day 14, two weeks after stopping the use of the supplement, there was still evidence of liver dysfunction. More importantly, complete resolution of the hepatopathy was detected on Day 40. Recovery has also been shown to be possible in mice following one month of exposure to a sublethal concentration of MC-LR [30, 31]. Similar to the dog in the current case, it took more than 30 days for complete resolution of the biochemical alterations induced by the exposure to MC-LR in mice. Interestingly, hepatic lesions consisting of hepatocellular disarray and loss of hepatic architecture were for the most part reversed 60 days after a month-long exposure to MC-LR in mice. Although biopsy of the liver was not attempted in the current case during or after exposure to MC-LR and MC-LA for comparison, we can only assume structural recovery also occurred in this dog. Had the dog continued to receive the dietary supplement, long-term health effects following chronic exposure may have ensued such as nephrotoxicity and tumor promotion which have been documented in long-term administration studies [32, 33].

At the time of presentation, additional differentials such as infectious (leptospirosis, bacterial), non-infectious inflammatory (hepatitis, cirrhosis), copper associated hepatopathy, toxin exposure, and neoplasia were all considered. Complete resolution of the patient’s hepatopathy following hospitalization suggested that neoplasia, copper associated hepatopathy, and non-infectious inflammatory differentials were unlikely. Historical initiation of the blue-green algae supplement prior to development of clinical and biochemical abnormalities and subsequent testing of the supplement confirming the presence of hepatotoxic microcystins confirmed the diagnosis of hepatopathy due to toxin exposure. Blue-green algae toxicosis is usually a result of drinking contaminated water or accidently drinking water while swimming [18, 34]. This is the first documented case of blue-green algae intoxication in a dog from ingestion of a dietary supplement. Blue-green algae products and other dietary supplements are regulated by the FDA; however, under the 1994 DSHEA, manufacturers of dietary supplements can market their products without having to demonstrate product safety [35]. Therefore, the dietary supplement industry is largely self-regulated, relying on information provided by the actual manufacturers or to the FDA about contamination or adverse events after use. Many consumers and animal owners believe that these products can only be sold if they are safe for use. Unfortunately, the opposite has been demonstrated in several studies showing the contamination of blue-green algae dietary supplements with microcystins [5, 7, 9]. The absolute purity of any blue-green algae product, including the absence of contamination with hepatotoxic microcystins, cannot be assured and thus, consumers and pet owners should err on the side of caution when purchasing and using these products.

## Conclusions

To the authors’ knowledge, this is the first case report of microcystin intoxication in a dog after using a commercially available blue-green algae dietary supplement. Consumers as well as veterinarians should recognize the potential harm in using these supplements. If adverse effects are noted, cessation of the product is recommended along with supportive care. In addition, more prudent oversight of dietary supplement use is recommended for our companion animals to prevent adverse events/intoxications.

## Abbreviations

CRI: Constant rate infusion
h: hour
IV: Intravenous
OU: Both eyes
PO: Orally
q: every
s: sec
SC: Subcutaneous
